# Another prospective study on the safety of ondansetron for nausea and vomiting of pregnancy: addressing ongoing concerns

**DOI:** 10.3389/fphar.2026.1783632

**Published:** 2026-03-10

**Authors:** Irina Tolchinsky, Maayan Beckinshtein, Elkana Kohn, Rana Cohen, Tal De-Haan, Tomer Ziv-Baran, David Stepensky, Itai Gueta, Matitiahu Berkovitch, Maya Berlin

**Affiliations:** 1 Department of Clinical Biochemistry and Pharmacology, Faculty of Health Sciences, Ben-Gurion University of the Negev, Beer Sheva, Israel; 2 Clinical Pharmacology and Toxicology Unit, Shamir (Assaf Harofeh) Medical Center, Zerifin, Affiliated to the Gray Faculty of Medical & Health Sciences, Tel-Aviv University, Tel Aviv, Israel; 3 School of Public Health, the Gray Faculty of Medical & Health Sciences, Tel Aviv University, Tel Aviv, Israel; 4 Department of Internal Medicine F, The Clinical Pharmacology Unit, Tel-Aviv Medical Center, Faculty of Medicine, Tel-Aviv University, Tel Aviv, Israel; 5 The Andy Lebach Chair of Clinical Pharmacology and Toxicology, The Gray Faculty of Medical & Health Sciences, Tel-Aviv University, Tel Aviv, Israel

**Keywords:** EMA (european medicines agency), ENTIS (european network of teratology information services), HG (hyperemesis gravidarum), major malformations, NVP (nausea and vomiting of pregnancy), ondansetron

## Abstract

**Background:**

The safety of ondansetron for nausea and vomiting in pregnancy (NVP) remains a subject of ongoing debate, driven by conflicting study results and a disagreement between the European Medicines Agency (EMA) and the European Network of Teratology Information Services (ENTIS) regarding its potential fetal risks.

**Objective:**

To assess the safety of using ondansetron for NVP and its possible association with major birth defects and neonatal outcomes.

**Methods:**

A prospective cohort study was conducted based on structured telephone follow-ups of women with NVP who contacted the NVP Helpline at a teratology information service (TIS) at the Shamir Medical Center between April 2014 and April 2018. Exposure and clinical characteristics were recorded during the initial consultation, and pregnancy and neonatal outcomes were ascertained after the estimated date of delivery using a validated questionnaire. Women treated with ondansetron formed the exposed group; controls did not receive ondansetron but could use other NVP therapies. Subgroup analyses explored the dose and frequency of use.

**Results:**

The analysis included 260 women (137 exposed; 123 controls). Baseline characteristics were broadly comparable, except for higher NVP severity in the ondansetron group, reflected by higher PUQE scores (9.86 ± 2.66 vs. 8.59 ± 2.49; p = 0.001) and more frequent antacid use (p = 0.001). Live birth rates (95.6% vs. 94.3%) and birth weights did not differ between groups. No differences were found in neonatal complications. Major congenital malformations were comparable between groups (5.1% vs. 4.1%; p = 0.695), with no consistent pattern identified.

**Conclusion:**

In this prospective cohort, ondansetron exposure was not associated with increased risk of adverse neonatal outcomes or major congenital malformations. The sample size limits the detection of very rare outcomes, and larger studies remain warranted.

## 1 Introduction

Nausea and vomiting of pregnancy (NVP) affect up to 85% of pregnant women. While mild to moderate NVP is not linked to serious maternal or fetal harm, it can significantly impair maternal quality of life. Severe NVP and hyperemesis gravidarum (HG) pose risks to maternal health and may impact fetal development (Bustos et al., 2017). However, concerns about potential fetal risks from NVP treatments lead many women to hesitate to use medications (Matsui, 2012). Delaying or avoiding treatment may increase risks for both the mother and the fetus. Most medications used for NVP have been shown to be safe during pregnancy (Goodwin, 2008).

The safety of ondansetron, a 5-HT3 receptor antagonist, when used during the first trimester of pregnancy remains controversial due to conflicting study outcomes. While some studies suggest that ondansetron does not increase the risk of cardiac and/or orofacial malformations (Masarwe et al., 2023; Sakran et al., 2021; Huybrechts et al., 2020), other studies do not confirm these findings or only partially confirm them (Huybrechts et al., 2018; Picot et al., 2020).

In 2019, the European Medicines Agency (EMA) advised against the use of ondansetron in the first trimester, citing possible links to orofacial and cardiac malformations (European Medicines Agency, 2019). However, the European Network of Teratology Information Services (ENTIS) disagreed, citing large-scale studies and supporting ondansetron as a second-line treatment for NVP (ENTIS, 2019).

Similarly, American College of Obstetricians and Gynecologists (ACOG) guidance includes ondansetron as a third-line pharmacological therapy for NVP, whereas the first-line therapy is reserved for doxylamine + pyridoxine combinations (ACOG, 2018).

Given the inconclusive evidence about ondansetron’s safety and the conflicting recommendations among organizations, this study aimed to investigate the effects of ondansetron on mothers with NVP and their newborns.

## 2 Materials and methods

### 2.1 Study design and study population

This study was conducted at the Zerifin Teratology Information Service (TIS) of the Clinical Pharmacology and Toxicology Unit at Shamir Medical Center, a tertiary referral hospital in Israel. Data were collected prospectively from women nationwide who contacted the TIS helpline for counseling between April 2014 and April 2018.

The first encounter occurred when a pregnant woman called the center for NVP advice. During the consultation, medical history, demographic details of the current and previous pregnancies, medications, and NVP symptoms were recorded.

This study utilized a pre-existing data repository from women who sought counseling and support from the TIS and consented to follow-up. Exposure information was collected during pregnancy at the time of counseling, and pregnancy and neonatal outcomes were actively ascertained after the estimated date of delivery through structured telephone interviews with the mothers. The protocol was approved by the institutional review board (ASF-0001-20); verbal informed consent was obtained at the beginning of the telephone follow-up call.

A telephone follow-up call was conducted after the expected date of delivery to collect information about the delivery process, complications during delivery, diseases of the offspring, and congenital malformations. The information was collected using a validated questionnaire (Schaefer et al., 2008; Winterfeld et al., 2013; Dao et al., 2022) (Supplementary File 1). Participants were classified as “lost to follow-up” after five unsuccessful contact attempts made on different days and times. Exclusion criteria included: refusal to participate, incomplete questionnaire responses, twin pregnancies, and long-term medications with known teratogenicity or established pregnancy risks.

### 2.2 Data measures

The primary objective was to evaluate the association between maternal ondansetron exposure during the first trimester and the risk of major congenital malformations and neonatal outcomes in the offspring.

The defined daily dose (DDD) for ondansetron is 16 mg, defined as the assumed average maintenance dose per day for a drug used for its main indication in adults for both oral and parenteral administration (WHO, 2024).

Daily dose of ondansetron (high: >16 mg; low: ≤16 mg), duration, and frequency of use (continuous for ≥1 week; or as-needed use) were recorded. Additional obstetric and neonatal outcomes were recorded. Major congenital malformations were analysed in two different ways: the EUROCAT classification (EUROCAT, 2013) and blinded expert review by two teratology specialists.

Secondary variables included maternal comorbidities, concurrent medication use, folic acid, iron and other supplement use, exposure to addictive substances during pregnancy, previous miscarriages, duration of labor, delivery method (vaginal or cesarean), breastfeeding, infant vaccination status, and infant weight at follow-up.

Comparing the background characteristics of the study group and the control group allowed assessment of potential selection bias, which may influence the results. Variability between groups was examined across different parameters: age, BMI (Body Mass Index), years of education, number of pregnancies and children, miscarriages, use of IVF (*In Vitro* Fertilisation) medications, PUQE (Pregnancy-Unique Quantification of Emesis) score, pregnancy checkups, maternal chronic disease, and harmful habits.

### 2.3 Statistical analysis

Categorical variables were described using frequency and percentage. Continuous variables were examined for normal distribution using a histogram and a Q-Q plot. Normally distributed continuous variables were described using the mean and standard deviation, while non-normally distributed variables were described using the median and interquartile range. Categorical variables were analyzed using a chi-squared test or Fisher’s exact test. Continuous variables were compared between groups using the independent-samples t-test, and non-normally distributed variables were analysed using the Mann-Whitney test or Kruskal–Wallis test when appropriate. Since the incidence of outcomes in the study was very low, and to maintain a ratio of 10 outcomes per variable, it was not possible to perform a multivariable analysis.

All tests were two-sided with a significance of p < 0.05. SPSS software (IBM SPSS Statistics for Windows, Version 27, IBM Corp, Armonk, New York, United States) was used for all analyses (IBM Corp, 2020).

## 3 Results

Telephone interviews were attempted with 388 women who contacted the NVP helpline between April 2014 and 2018. Of these, 82 did not respond, 38 declined participation, and 3 began but did not complete the interview. A total of 265 women were included. Five were later excluded due to twin pregnancies (n = 4) or chronic use of bezafibrate (n = 1), leaving 260 women in the final analysis. Among them, 137 were exposed to ondansetron (study group), and 123 were not (control group).

### 3.1 Background characteristics

There were no significant differences between the two groups in most baseline characteristics, except for the PUQE score. The study group reported a higher PUQE score at consultation (9.86 ± 2.66) compared to the control group (8.59 ± 2.49) (p = 0.001), indicating more severe NVP symptoms. Severe NVP, defined as PUQE ≥13, was more frequent in the study group (18/130, 13.8%) than in controls (9/116, 7.8%), although this difference did not reach statistical significance (p = 0.13).

Mean maternal age in both groups was around 31 years, the median BMI was approximately 22, and education levels were similarly high. The number of pregnancies, parities, and miscarriages did not differ significantly. Additionally, both groups showed no significant differences in the use of IVF medications, frequency of pregnancy checkups, presence of chronic disease, and harmful habits (see Table 1).

**TABLE 1:** Background maternal characteristics.

Variable	Study group ondansetron exposed n = 137	Control group n = 123	P-value
Maternal age at first contact, mean ± SD, y	31.2 ± 5.5	31.3 ± 4.8	0.849
BMI PPW, median (IQR)	21.8 (20.4–25.2)	21.9 (20.1–24.5)	0.685
Education, mean ± SD, y	15.7 ± 2.4	15.8 ± 2.6	0.709
Gravidity, median (IQR)	3 (2–4)	2 (1–4)	0.452
Primigravida, n (%)	37 (31.4%)	34 (25.6%)	0.31
Parity, median (IQR)	1 (0–2)	1 (0–2)	0.453
Miscarriages, median (IQR)	0 (0–1)	0 (0–0)	0.984
Therapeutic abortions, median (IQR)	0 (0–0)	0 (0–0)	0.634
IVF medications, n (%)	14 (10.2)	7 (5.7)	0.187
PUQE score at consult date, mean ± SD	9.86 ± 2.66	8.59 ± 2.49	0.001
Pregnancy checkups, n (%)	93 (69.4)	93 (78.2)	0.115
Endocrine disease, n (%)	14 (10.2)	7 (5.7)	0.181
Cardiovascular problems, n (%)	2 (1.5)	0 (0)	0.500
CNS, n (%)	45 (32.8)	32 (26.0)	0.228
Skin problems, n (%)	7 (5.1)	5 (4.1)	0.689
Diabetes, n (%)	8 (5.8)	8 (6.5)	0.824
Nose ear throat, n (%)	3 (2.2)	0 (0)	0.249
Infectious disease, n (%)	1 (0.7)	3 (2.4)	0.347
Digestive system problems, n (%)	11 (8.0)	8 (6.5)	0.637
Urinary disease, n (%)	1 (0.7)	3 (2.4)	0.347
Hematologic problems, n (%)	14 (10.2)	7 (5.7)	0.181
Skeletal muscular problems, n (%)	6 (4.4)	4 (3.3)	0.753
Respiratory system problems, n (%)	4 (2.9)	5 (4.1)	0.739
Allergy, n (%)	18 (13.1)	14 (11.4)	0.667
Alcohol use during pregnancy, n (%)	2 (1.5)	3 (2.4)	0.670
Smoking during pregnancy, n (%)	2 (1.5)	3 (2.4)	0.670
Cannabis during pregnancy, n (%)	4 (2.9)	1 (0.8)	0.373

IQR, interquartile range; SD, standard deviation; BMI PPW, Body Mass Index Pre-Pregnancy Weight; IVF, in vitro fertilisation.

### 3.2 Medication use during pregnancy

Table 2 summarizes the most frequently used medications during pregnancy in both groups, classified by the ATC (Anatomical Therapeutic Chemical) classification (WHO, 2024), based on the chemical group of each medication.

**TABLE 2:** Most frequent medications that were used during pregnancy.

Variable	Study group ondansetron exposed N = 137	Control group N = 123	P-value
Other NVP drugs, n (%)	116 (84.7)	104 (84.6)	0.979
Psychotropic medications, n (%)	9 (6.6)	14 (11.4)	0.172
Antacid medications, n (%)	43 (31.4)	18 (14.6)	0.001
Thyroid medications, n (%)	5 (3.6)	4 (3.3)	>0.999
Iron IV, n (%)	8 (5.8)	5 (4.1)	0.512
Folic acid, n (%)	94 (70.1)	94 (77.7)	0.172
Iron, n (%)	48 (35.8)	52 (43)	0.243
Prenatal (multi vitamin for pregnant women), n (%)	41 (30.6)	45 (37.2)	0.266

The variable “Other NVP drugs” includes medications considered safe for NVP (Bustos et al., 2017; Koren, 2017), such as dimenhydrinate, doxylamine succinate + pyridoxine hydrochloride, metoclopramide.

Most women in the study group were exposed to other NVP medications in addition to ondansetron (116 of 137). There were no significant differences between the groups in most medications and supplements, except for antacid medications (p = 0.001). This variable includes H2-receptor antagonists, PPIs (proton pump inhibitors), antacids (calcium carbonate), and calcium with magnesium complexes.

### 3.3 Characteristics of ondansetron use among the study group

Among the study group, 2 women were exposed to both ondansetron and granisetron during pregnancy, and one used granisetron only. As both belong to the 5-HT3 receptor antagonist class, granisetron use was considered equivalent to ondansetron.

Most women (76.7%, n = 102) were exposed to a low daily dose of the medication (≤16 mg), and 72.3% (n = 94) used ondansetron daily for at least 1 week.

In four cases, the daily dose was unknown, and in seven, the duration was unclear; these women were excluded from dose-duration analyses.

In most cases, ondansetron was initiated in the 8th week and continued until the 20th week of pregnancy. Five women experienced NVP almost until the end of their pregnancies.

Out of 137 women, 118 (86.1%) were exposed to ondansetron during the first trimester, and 77 (56.2%) of them continued into the later trimesters.

Among the 31 women taking high doses (>16 mg), 15 (48.4%) reported continuous use (see Table 3).

**TABLE 3:** Characteristics of ondansetron use among the study group.

Variable	Study group ondansetron exposed N = 137
Ondansetron high dose, n (%)	31 (23.3)
Ondansetron low dose, n (%)	102 (76.7)
Sporadic use of ondansetron, n (%)	36 (27.7)
Permanent use of ondansetron, n (%)	94 (72.3)
Start week of ondansetron use, median (IQR)	8 (7–10)
End week of ondansetron use, median (IQR)	20 (12–37)

IQR, interquartile range.

### 3.4 Pregnancy and neonatal outcomes

As shown in Table 4, there was no significant difference between the study group and the control group regarding live birth rates (n = 131, 95.6% vs. n = 116, 94.3%). Additionally, there were no differences in birth weight or sex parameters between the groups. A statistically significant difference was observed in the week of birth (p = 0.043), with a median of 39 weeks in the study group versus 40 weeks in the control group.

**TABLE 4:** Obstetric outcomes of newborns.

Variable	Study group ondansetron exposed N = 137	Control group N = 123	P-value
Miscarriage <20 weeks, n (%)	2 (1.5)	3 (2.4)	0.670
Fetal death ≥20 weeks, n (%)	0 (0)	1 (0.8)	0.473
Elective termination, n (%)	3 (2.2)	3 (2.4)	>0.999
Stillbirth, n (%)	1 (0.7)	0 (0)	>0.999
Live birth, n (%)	131 (95.6)	116 (94.3)	0.628
Sex, n (%)- female	72 (55)	67 (57.8)	0.658
Delivery method – vaginal, n (%)	96 (89.7%)	119 (94.4%)	0.27
C/S, n (%)	11 (10.3%)	7 (5.6%)	
Week of birth, median (IQR)	39 (38–40)	40 (39–41)	0.043
Weight of birth, mean ± SD, gr	3,218.05 ± 477.77	3,267.11 ± 449.96	0.414
Newborn complications at birth	27 (20.6)	31 (26.7)	0.258
APGAR 1 min, score 7, n (%)	2 (2.4)	0 (0)	0.156
APGAR 1 min, score 8, n (%)	1 (1.2)	3 (4)	​
APGAR 1 min, score 9, n (%)	80 (96.4)	70 (93.3)	​
APGAR 1 min, score 10, n (%)	0 (0.0)	2 (2.7)	​
APGAR 5 min, score 9, n (%)	7 (8.9)	5 (7)	0.682
APGAR 5 min, score 10, n (%)	72 (91.1)	66 (93)	​
Complications symptoms of the newborn at birth	3 (2.3)	0 (0)	0.250
Medical intervention as a result of newborn perinatal complications	4 (3.1)	3 (2.6)	>0.999
Major birth defects, n (%)	7 (5.3)	5 (4.3)	0.695
Breastfeeding, n (%)	114 (87.0)	92 (79.3)	0.241
Breastfeeding partial, n (%)	1 (0.8)	3 (2.6)	
Breastfeeding until 3-month, n (%)	18 (15.8)	11 (11.8)	0.408
Breastfeeding until 3–6 months, n (%)	39 (34.2)	27 (29.0)	
Breastfeeding until more than 6 months, n (%)	57 (50.0)	55 (59.1)	
Newborn vaccination, n (%)	116 (89.2)	95 (81.9)	0.100

IQR, interquartile range; SD, standard deviation; C/S, caesarean section.

Neonatal complications during birth, including fetal distress, aspiration of meconium-stained amniotic fluid, and need for specialized monitoring, showed no significant differences between the groups (p = 0.258).

Most newborns in both groups achieved high APGAR scores at the first and fifth minutes of life, with no statistical differences.

In the study group, three newborns (2.3%) exhibited complication symptoms immediately after delivery: one with hypoglycaemia, another with a change in heart rate, and the third with distress that required intensive care hospitalization. These were not statistically different from the control group.

### 3.5 Neonatal major birth malformations

No statistically significant difference was found between groups regarding major neonatal malformations (p = 0.695) (Table 4). Twelve cases were reported: 7 in the study group (5.1%) and 5 in the control group (4.1%). Table 5 shows no consistent pattern of specific malformations in the study group.

**TABLE 5:** Neonatal major birth malformations.

Study group ondansetron exposed N = 137	Control group N = 123
One testicle (birth week- 39)	One kidney (birth week-40)
Femoral deformity (birth week- 39)	Inguinal hernia (birth week-35)
Cleft lip (birth week- 38)	Haemangioma (birth week-32)
Cryptorchidism (birth week- 38)	Thanatophoric dysplasia (elective termination, week-17)
Left cheek haemangioma (birth week- 38)	Brain cysts (birth week-41)
Pelvic kidney (birth week-40)	
Inguinal hernia (birth week- 38)	

### 3.6 Obstetric and neonatal outcomes by ondansetron dose

To assess the association between ondansetron dosage and maternal or neonatal complications, participants were divided into three groups: no exposure, low dose (≤16 mg), and high dose (>16 mg). As shown in Table 6, most outcomes showed no statistically significant differences between the no-exposure and low-dose groups (a). Significant differences were found when comparing the no-exposure group to the high-dose group (b), and the low-dose group to the high-dose group (c), specifically in neonatal complications immediately after birth (p = 0.015).

**TABLE 6:** Obstetric and neonatal outcomes by ondansetron dose category.

Variable	Non exposed to ondansetron N = 123	High dose ondansetron N = 31	Low dose ondansetron N = 102	P-value*	a = non/low dose b = non expose/high c = low/high
Delivery mother complications, n (%)	5 (4.3)	2 (6.5)	7 (7.1)	0.671	​
Newborn complications at birth	31 (26.7)	8 (25.8)	18 (18.2)	0.312	​
Complication symptoms right after birth	0 (0)	2 (6.5)	0 (0)	0.015	b, c
Newborn perinatal complications	3 (2.6)	1 (3.2)	2 (2.0)	0.863	​
Birth week above 37	112 (96.6)	30 (96.8)	99 (100)	0.121	​
Major birth defects	5 (4.3)	1 (3.2)	6 (6.1)	0.915	​

*P-values represent the global comparison across the three groups.

Where significance was reached, specific pairwise differences are denoted as follows: (a) Non-exposed vs. Low dose; (b) Non-exposed vs. High dose; (c) Low dose vs. High dose.

### 3.7 Obstetric and neonatal outcomes by ondansetron use frequency

Table 7 shows no significant differences between sporadic use, continued use of ondansetron, and no exposure at all, in terms of obstetric outcomes, maternal and newborn complications, birth week, or the occurrence of major malformations.

**TABLE 7:** Obstetric and neonatal outcomes by ondansetron use frequency.

Variable	Non exposed to ondansetron N = 123	Sporadic use N = 36	Continuous use N = 94	P-value*
Delivery mother complications, n (%)	5 (4.3)	0 (0)	8 (9.0)	0.127
Newborn complications at birth	31 (26.7)	5 (14.3)	20 (22.5)	0.304
Complication symptoms right after birth	0 (0)	0 (0)	1 (1.1)	0.517
Newborn perinatal complications	3 (2.6)	0 (0)	3 (3.4)	0.861
Birth week above 37	112 (96.6)	34 (97.1)	89 (100)	0.142
Major birth defects	5 (4.3)	1 (2.9)	6 (6.7)	0.712

*P-values represent the global comparison across the three groups.

## 4 Discussion

### 4.1 Principal findings

In this prospective cohort study of women with NVP, no statistically significant differences were observed in major neonatal outcomes between pregnancies exposed to ondansetron and those unexposed. Specifically, rates of live birth, birth weight, APGAR scores, neonatal complications, and major congenital malformations were comparable between groups. The overall rate of major malformations was within the expected background range of 3%–5% and did not differ significantly between exposed and control groups, nor did it show a consistent malformation pattern.

A slight but statistically significant difference in delivery week was observed (p = 0.043): median 39 weeks (IQR 38–40) vs. 40 weeks in the control group (IQR 39–41); however, this difference remained within the normal term range.

Higher PUQE scores in the exposed group reflected more severe NVP, supporting clinical indication rather than exposure-related bias. In line with this, severe NVP (PUQE ≥13) was more frequent in the exposed group (18/130, 13.8%) than in controls (9/116, 7.8%), although this difference did not reach statistical significance (p = 0.13). The study group experienced more severe NVP, with 84.7% (n = 116) using other NVP medications in addition to ondansetron, compared to the control group, where 15.4% (n = 19) did not require any NVP medications.

When stratifying by dose, a statistically significant difference emerged in the occurrence of neonatal complications immediately after birth among high-dose ondansetron users compared with low-dose and unexposed groups. However, the absolute number of affected cases was very small, limiting clinical interpretability. No association was found between ondansetron exposure, dose, or frequency of use and the risk of major congenital malformations.

The higher PUQE scores observed in the ondansetron group are consistent with clinical practice, as ondansetron is typically reserved for women with moderate-to-severe NVP or HG (Bustos et al., 2017; Goodwin, 2008). Increased antacid use among exposed women likely reflects more severe gastrointestinal symptoms, including reflux, which commonly accompany severe NVP and HG (Body and Christie, 2016).

The absence of significant differences in neonatal outcomes such as live birth rate, birth weight, APGAR scores, and immediate neonatal complications aligns with several prior prospective and retrospective studies reporting no increased overall risk associated with ondansetron use during pregnancy (Masarwe et al., 2023; Sakran et al., 2021; Huybrechts et al., 2020; Pasternak et al., 2013). The slight reduction in gestational age at delivery observed in the exposed group is consistent with prior literature, suggesting that severe NVP itself (Ayyavoo et al., 2014), rather than antiemetic exposure, may be associated with modest differences in gestational duration (Pasternak et al., 2013; Cao et al., 2022).

The lack of association between ondansetron exposure and specific congenital malformation patterns is in agreement with multiple observational studies and meta-analyses that found no consistent increase in cardiac or orofacial malformations (Masarwe et al., 2023; Sakran et al., 2021; Huybrechts et al., 2020; Picot et al., 2020; Cao et al., 2022). At the same time, these findings contrast with certain registry-based studies that reported small increases in specific defects, highlighting the ongoing inconsistency in the literature and the importance of prospective data collection (Huybrechts et al., 2018). The percentage of severe malformations in newborns in this study was 4.6%, with no statistically significant difference between the study and control groups and no consistent pattern of major malformations. This aligns with the general 3%–5% rate of congenital malformations (Finnell, 1999). No evidence linked ondansetron to increased cardiac or orofacial malformations. However, given EMA warnings and one cleft lip case in our study, it is critical to consider the plausibility of a causal association.

This case involved a 34-year-old woman (BMI 26.84) with high education, fibromyalgia, no harmful habits, and a history of miscarriage. She used folic acid preconception, but did not continue it during pregnancy, and received fertility medications. Her PUQE score was 10 at the 15th week of pregnancy and 8 by the 17th week; genetic and routine prenatal tests were normal. She used a low dose of ondansetron (8 mg) daily from week 4–38, received several intravenous ondansetron doses during the 11th to 13th weeks, and a few doses of metoclopramide IV in the 15th week. The male infant was delivered by emergency cesarean section at 38 weeks due to cord entanglement, weighed 3,200 g, and had a cleft lip with cyanosis and respiratory distress.

The absence of similar cases in the exposed group, combined with the presence of other potential confounders, makes the causal role of ondansetron uncertain. A cleft lip with or without a cleft palate is a common congenital malformation, often influenced by genetic and environmental risk factors (Khan and CS, 2020).

### 4.2 Clinical and research implications

From a clinical perspective, these findings provide additional reassurance regarding the short-term neonatal safety of ondansetron when used for NVP, including first-trimester exposure. The comparable rates of major malformations and neonatal complications between exposed and unexposed groups support its continued consideration as a second- or third-line therapy in women with refractory symptoms, in line with ENTIS and ACOG guidance (ENTIS, 2019; ACOG, 2018).

The isolated observation of neonatal complications among a small number of high-dose users should be interpreted with caution due to limited sample size and low absolute event rates. While this finding raises the possibility of a dose-related effect, its clinical significance remains uncertain and does not support a change in current prescribing practices.

The single case of cleft lip observed in the exposed group occurred in the presence of multiple potential confounding factors, including fertility treatment, discontinuation of folic acid supplementation, intravenous antiemetic exposure, and obstetric complications. Cleft lip with or without cleft palate is among the most common congenital malformations and is known to be influenced by multifactorial genetic and environmental contributors (Khan and CS, 2020). The absence of a consistent pattern of malformations and the lack of a dose–response relationship argue against a causal association.

Overall, these data support the ENTIS position regarding the safety of Ondansetron use during pregnancy for both the mother and the newborn (ENTIS, 2019). These findings are particularly important in challenging clinical situations such as HG, in which ondansetron may play a critical therapeutic role.

Despite growing evidence supporting the relative safety of ondansetron, important unanswered questions remain. Larger prospective studies are needed to more precisely evaluate rare outcomes, including specific congenital malformations and potential dose-related neonatal effects. Such studies should be sufficiently powered to allow multivariable analyses addressing confounding by indication, disease severity, and concomitant medication use (Picot et al., 2020; Cao et al., 2022).

Further research into the pharmacokinetics of ondansetron during pregnancy and placental transfer at different gestational stages may help clarify the biological plausibility for reported associations, given the documented placental passage of the drug (Siu et al., 2006). Long-term neurodevelopmental follow-up studies may also be valuable to address outcomes beyond the perinatal period (Ayyavoo et al., 2014).

### 4.3 Strengths and limitations

A major strength of this study is its prospective design, with systematic data collection beginning at the first consultation and extending through postnatal follow-up. The inclusion of women actively seeking counseling for NVP ensured detailed documentation of symptom severity, medication use, and pregnancy course, which is often lacking in registry-based studies.

The study population largely consisted of women with moderate-to-severe NVP, making the findings particularly relevant to clinical scenarios in which ondansetron is most frequently considered (Bustos et al., 2017; Goodwin, 2008). The dual assessment of congenital malformations using EUROCAT classification and blinded expert review strengthens outcome validity (EUROCAT, 2013).

Limitations include reliance on self-reported data, which may introduce recall bias, particularly for lifestyle factors such as smoking or alcohol use (Matsui, 2012). However, women with persistent NVP are more likely to remember their symptoms and medication use. Additionally, major neonatal malformations are typically visible, minimizing recall bias in this domain.

The relatively small sample size limits statistical power for rare outcomes and subgroup analyses by dose or frequency and precludes multivariable modeling.

### 4.4 Conclusions

In this prospective cohort study, ondansetron use for NVP was not associated with an increased risk of adverse neonatal outcomes or major congenital malformations compared with unexposed pregnancies. However, the small sample size may have limited its ability to detect a true effect. Larger-scale studies are necessary to validate these findings, especially in evaluating the impact of low versus high doses of ondansetron on obstetric outcomes. Until such data are available, ondansetron remains a reasonable therapeutic option for women with severe NVP when clinically indicated.

## Data Availability

The raw data supporting the conclusions of this article will be made available by the authors, without undue reservation.
