# Supplementary material for: Another prospective study on the safety of ondansetron for nausea and vomiting of pregnancy: addressing ongoing concerns
Source: Front Pharmacol. 2026 Mar 10;17:1783632. doi: 10.3389/fphar.2026.1783632 (PMC13008973; doi:10.3389/fphar.2026.1783632)
Supplement: Supplementary file 1 [file DataSheet1.pdf]

*Supplementary Material*

**Another Prospective Study on the Safety of Ondansetron for Nausea and Vomiting of Pregnancy: Addressing Ongoing Concerns**

Irina Tolchinsky[1], Maayan Beckinshtein[1], Elkana Kohn[2], Rana Cohen[2], Tal De-Haan[2], Tomer Ziv-Baran[3], David Stepensky[1], Itai Gueta[4], Matitiahu Berkovitch*[2,5], and Maya Berlin[2]

*Clinical Pharmacology Unit*
*Shamir Medical Center*

# Follow-up Form

**File number**:__________________________ Date of follow up:__________________________.

**Verbal permission to participate in the study: Yes ☐ No ☐**

## General Information

Mother's name: ___________________________ Age: _____

Tel: home______________

When consulting the TIS - Gestational age ____wks G___ P___ SA___ TA___

Years of schooling____ Maternal profession ______________

Number of children: ___ Previous malformations: _______________________________

| | |
|---|---|
| **LMP:** | Pregnancy of interest outcome |
| Place of the spoken child: ___ | Live Birth ☐ |
| **D.O.B:** | **Miscarriage (<20wks) ☐** |
| **At the time of follow up: G___ P___ SA___ TA___ Ectopic___ Molar___Other___** | **Fetal Death (>=20wks) ☐** |
| | **Elective Abortion ☐** |

## Diseases Complications during pregnancy

| | | | | | |
|---|---|---|---|---|---|
| **Endocrine (e.g.thyroid)** | **Dx:** | | **Infectious Diseases** | **Dx:** | |
| **Cardiovascular (e.g. blood pressure)** | **Dx:** | | **Gastro-Intestinal (e.g. heartburn)** | **Dx:** | |
| **Central Nervous System (e.g. epilepsy, headache, migraine)** | **Dx:** | | **Genito-Urinary (e.g. yeast infections)** | **Dx:** | |
| **Dermatology (e.g. rash)** | **Dx:** | | **Hematology (e.g. anemia)** | **Dx:** | |
| **Diabetes** | **Dx:** | | **Muscular-Skeletal (e.g. arthritis, fractures)** | **Dx:** | |
| **Ears, Eyes, Nose, Throat (e.g. sinuses, allergies)** | **Dx:** | | **Respiratory (e.g. asthma)** | **Dx:** | |
| **Psychiatric Disorders (e.g. depression, anxiety)** | **Dx:** | | **Operations** | **Dx:** | |
| **Other** | **Dx:** | | **Other** | **Dx:** | |

Details:__________________________________________________________________

_________________________________________________________________________

_________________________________________________________________________

_________________________________________________________________________

## Drugs during pregnancy:

| MEDICATION OF INTEREST | INDICATION | START | STOP | DOSE /FREQ/ # OF DAYS DRUG WAS TAKEN | SIDE EFFECTS |
|---|---|---|---|---|---|
| | | Before pregnancy ☐<br>T1______wks<br>T2______wks<br>T3______wks | T1______wks<br>T2______wks<br>T3______wks<br>Ongoing ☐ | ______ mg X<br>______times per day X<br>_______ days<br><br>Other regiment:<br>_______________________ | |
| | | | | | |

**Please record all medications used during pregnancy (prescription or over-the-counter OTC)**

| Medication | START: | STOP: | ONGOING: | AMOUNT: |
|---|---|---|---|---|
| | | | | |
| | | | | |

## Exposures during pregnancy

| | START: | STOP: | ONGOING: | AMOUNT: |
|---|---|---|---|---|
| **Alcohol** | | | | |
| **Tobacco** | | | | |
| **Marijuana** | | | | |
| **Cocaine** | | | | |
| **Radiation** | | | | |
| **Hyperthermia** | | | | |

## Ultrasound and other tests:

| Test | Gestational age | Indication (routine / specific) | Results |
|---|---|---|---|
| Nuchal Translucency Screening | | | |
| Alpha fetoprotein / The Combined Test- First Trimester Screening | | | |
| Integrated Test | | | |
| level II US | | | |
| Amniocentesis/ Chorionic villus sampling (CVS) | | | |
| Fetal echocardiography | | | |
| level II US late | | | |

frontiers

**Delivery Information**

**If miscarriage, fetal death or therapeutic abortion**:
at how many weeks ________ months ___________ any defects detected (describe) ___________________________________

**If live birth:** **boy_____ girl_____twins_____**
Child's first name: ______________________________ Birth place: ____________________
Child's DOB: _________________________________ Gestational age of birth: _____
Birth weight: ____________________

- Length of labours (h): ____________
- Delivery method: [ ] vaginal (vertex, breech)
  [ ] Vacuum
  [ ] Forceps (low, mid, high)
  [ ] C/S (repeat / emergency)
- Premature rupture of membranes: yes [ ] no [ ]
- Delivery complications **(e.g. breech birth, cord wrapped around baby's neck, long labour, induced delivery, failure to progress, etc.)**
  ____________________________________________________________________________________________
  ____________________________________________________________________________________________
- Hemorrhage before or after delivery: yes [ ] no [ ]
- Transfusion required: yes [ ] no [ ]
- Drugs during birth: [ ] Anaesthetics ________________ [ ] Epidural [ ] Analgesic________________
  - Induction of birth [ ] no [ ] yes
  - Fetal monitoring [ ] no [ ] yes
  - Fetal distress [ ] no [ ] yes
  - APGAR scores: 1 min___ 5 min ___

- **Any complications with the baby at birth:** Yes ☐ No ☐
- **Did the complications appear right after birth or after a few days?** ___________________________
- **Was the baby in a special care nursery:** Yes☐ No ☐
- If yes, for how long: ________________days?
- **What symptoms did the baby have?**
- ☐ heart rate changes________________________________
- ☐ meconium staining (swallowed yes☐ no☐)____________
- ☐ difficulty breathing________________________________
- ☐ fetal distress_____________________________________
- ☐ low blood sugar___________________________________
- ☐ jaundice__________________________________________
- ☐ blue baby (cyanosis) ______________________________
- ☐ jitteriness_______________________________________
- ☐ poor muscle tone__________________________________

- ☐ other:________________________________________________
- **Medical intervention:** Yes☐ No☐
- If yes, what type (oxygen, monitoring heart rate, antibiotics, blood transfusion, respiratory support)____________
- Neonatal birth defects: [ ] no [ ] yes ________________________________________________ time of diagnosis of major malformation: __________________________________
- Neonatal health problems: [ ] no [ ] yes ________________________________________________ ________________________________________________________________________________________

frontiers

# INFANT HEALTH FOLLOWING DISCHARGE FROM THE HOSPITAL:

Any illnesses (e.g. ear infections, colds, etc.)? Yes ☐ No ☐

If yes, what kind: ____________________________________________

Did it require a hospital visit: Yes ☐ No ☐ Medication: Yes ☐ No ☐ Surgery: Yes ☐ No ☐

Home at (d):_____ Feeding (Breast / Bottle + times per day):______________________

Health since discharge: ______________________________________________________

Hospitalizations: ___________________________________________________________

Immunizations: [ ] no [ ] yes

Infant's age at the time of follow-up: ________________ months

Infant's weight _______________ kg at the time of follow-up ☐ or last MD visit ☐

Infant's height/length __________ cm at the time of follow-up ☐ or last MD visit ☐

Last MD visit: ______________________ (date or baby's age)

Was it a child's family doctor or pediatrician?
______________________________________________________________

**CONSENT**
**We would like to send a letter to your child's doctor to confirm medical details of this follow-up.**
**May we have your verbal permission to send this? ☐ No ☐Yes**

Date letter sent: Date letter received:

## Milestones

(For the following give the *age* of the infant when *first*:

| Events | Age | Normal values |
|---|---|---|
| Smiled | | 2 |
| Lifted head | | 3 |
| Sat unaided | | 6-8 |
| Crawled | | 8-10 |
| Stood | | 8-10 |
| First word (which was...) | | 8-12 |
| Walked unaided | | 12-15 |

**NVP Data**

Week of onset of nausea and vomiting: ______________________________

Severity of NVP: ___________________________________________

PUQE score at time of referral: ____________ Referral date: ___________________________

PUQE score at follow-up call: ____________ Follow-up call date: ______________________

Did you expect nausea and vomiting? Yes/No, __________________________________________

Was there NVP in a previous pregnancy? Yes/No,________________________________________
